# Prevalence, perceptions, and beliefs of university students about electronic cigarettes

**DOI:** 10.1590/1806-9282.20251056

**Published:** 2025-10-27

**Authors:** Camila Antunez Villagran, Felipe Teixeira Dias, Alcides Júnior Santos Lima, Cecília Gabrielle Lima Matos, Jefferson Traebert, Gabriel Oscar Cremona-Parma

**Affiliations:** 1 Universidade do Sul de Santa Catarina, Postgraduate Program in Health Sciences – Palhoça (SC), Brazil.; 2 Universidade de Rio Verde, Faculty of Nursing – Rio Verde (GO), Brazil.; 3 Universidade Estadual de Montes Claros, Postgraduate Program in Social Development – Montes Claros (MG), Brazil.; 4 Faculdades Integradas Padrão Guanambi Afya, Medicine Course – Guanambi (BA), Brazil.; 5 Universidade do Sul de Santa Catarina, Medicine Course – Palhoça (SC), Brazil.; 6 Universidade do Sul de Santa Catarina, Postgraduate Program in Administration – Palhoça (SC), Brazil.

**Keywords:** Electronic nicotine delivery systems, Students, Public health

## Abstract

**OBJECTIVE:**

The aim of this study was to estimate the prevalence of electronic cigarette use and to explore the perceptions and beliefs of university students regarding their use.

**METHODS:**

This is a cross-sectional study involving 437 students from higher education institutions of Guanambi, Bahia. A questionnaire based on five previous studies on electronic cigarette perceptions among students was applied, consisting of 25 questions addressing student characteristics, knowledge, and experimentation with electronic cigarettes. Bivariate analyses were performed to assess the relationship between electronic cigarette use, age, perceptions, and beliefs, using the chi-square test.

**RESULTS:**

The prevalence of electronic cigarette use was 39.2%, with no statistically significant difference between genders. The age of initiation was related to the places of use, predominantly parties and socialization environments. Individuals who used electronic cigarettes believed that they were more socially acceptable than conventional cigarettes and viewed them as a tool for smoking cessation.

**CONCLUSION:**

The results indicate a significant prevalence of electronic cigarette use among university students, with varied perceptions of its risks and benefits.

## INTRODUCTION

Tobacco use is a significant public health problem and a leading preventable cause of death, as highlighted by studies by the World Health Organization (WHO)^1,2^. Tobacco use is responsible for more than 8 million deaths annually, with a substantial proportion resulting from cardiovascular diseases, cancer, and respiratory diseases^3^. However, in recent years, a global paradox has emerged: while the consumption of conventional cigarettes has been decreasing due to long-term public policies, new forms of smoking have emerged^4^.

The introduction of e-cigarettes in 2003 marked a significant change in smoking habits, offering a new way to consume nicotine through electronic means^5,6^. Despite being considered less harmful than traditional cigarettes, knowledge about e-cigarettes remains limited, and many users are unaware of the potential risks associated with these devices^6^. International studies point to a growing trend in the use of these devices among adolescents and young adults. Research conducted in Europe and the USA reveals prevalence rates ranging from 20 to 40%, depending on age group and level of exposure to advertising^5–7^. Although e-cigarettes were initially perceived as an aid to smoking cessation, they pose health risks, including respiratory disorders, increased cardiovascular risks, and serious lung injuries, such as Electronic cigarette or Vaping-Associated Lung Injury (EVALI)^7^. The design of these devices incorporates features such as electromagnetic induction heating to produce efficient vaporization and consistent vaping experiences^8^.

These devices are currently primarily targeted at young people and, more worryingly, are marketed as harmless and safe. However, most contain nicotine levels that can vary significantly and even exceed those found in conventional cigarettes^4^.

Social media plays a crucial role as the primary platform for advertising and marketing these products. The reasons for their use range from robust marketing campaigns and ads with digital influencers to attractive flavors such as "cotton candy." Despite the resolutions of the National Health Surveillance Agency (ANVISA), online sales are easily accessible, and these products are also found in points of sale, parties, and nightclubs^4^.

This research stands out for offering an unprecedented analysis in the context of the semiarid Northeast, more specifically in a municipality in the interior of Bahia, where there is a scarcity of data on the use of electronic smoking devices among university students. This study aimed to estimate the prevalence of e-cigarette use and explore the perceptions and beliefs of university students in a municipality in Northeast Brazil.

## METHODS

A cross-sectional study was conducted in four higher education institutions in the municipality of Guanambi, located 796 km from the capital city of Salvador/BA, Brazil. The study included students regularly enrolled in these institutions, of both genders, with no age or academic period restrictions. All students meeting the inclusion criteria were invited to participate.

The inclusion criteria were: being 18 years or older, being a student at higher education institutions in Guanambi/BA, voluntarily agreeing to and signing the informed consent form, and having the physical, mental, and cognitive capacity to respond to the questionnaire. The exclusion criterion was not agreeing to sign the informed consent form.

To support the study, a closed, self-administered questionnaire without student identification was developed. The questionnaire was standardized and created by the authors based on the analysis of five previously published studies on students’ perceptions of electronic cigarettes. It consisted of 25 questions addressing student characteristics, knowledge, and experimentation with electronic cigarettes.

The study was conducted during the first semester of 2024 using printed questionnaires over a 60-day period through active searches in the higher education institutions of the municipality. The data collection was conducted in person during academic periods, where the authors explained the research objectives and ensured participant anonymity.

Volunteering students signed the informed consent form. The questionnaire was applied to a sample of 437 individuals distributed across the higher education institutions in the municipality, which had previously approved the study within their facilities.

Additionally, the study complied with the ethical principles outlined in Resolutions 466/2012 and 510/2016 of the Brazilian National Health Council for research involving human subjects^9^. Approved by the Research Ethics Committee of the State University of Bahia (UNEB), under protocol no. 77580824.0.0000.5109.

The sample size was calculated based on the formula for cross-sectional studies, considering a finite university population estimated at 5,000 students across the participating institutions, a 5% margin of error, a 95%CI, and an expected prevalence of 50% for electronic cigarette use. This resulted in a minimum required sample of 357 participants. To reduce potential losses and increase representativeness, a total of 437 students were included.

During data processing, questionnaires were manually reviewed at the time of collection to verify completeness. Missing or inconsistent data were excluded only from the specific statistical analyses in which the issue was identified, while valid portions of the questionnaire were retained for other analyses. No data imputation techniques were applied, and statistical tests considered only valid values for each variable analyzed.

All collected data were entered into an Excel® spreadsheet and transferred to R 4.4^10^ and RStudio 2024-4^11^ software for analysis. The characterization of electronic cigarette use was performed using proportions. Bivariate analyses of electronic cigarette use, age, perceptions, and beliefs were conducted using the chi-square test and t-test, with p<0.05 considered statistically significant.

## RESULTS

A total of 437 individuals were included in the study. The prevalence of electronic cigarette use was 39.2% (95%CI 34.4–43.8). Overall, no statistically significant difference was observed between genders (p=0.75). However, the prevalence was significantly higher among younger women compared to younger men in the age groups of 18–20 years (p=0.040) and 21–23 years (p=0.040).

When considering age alone, a significant difference was also observed in favor of younger individuals (p<0.001). The distribution by gender and age group is presented in Table 1.

**Table 1:** Use of electronic cigarettes by sex and age group. Guanambi/BA, 2024 (n=171).

Sex/age group		n	%
Male		78	45.6
	18–20 years	19	24.4
	21–23 years	34	43.6
	24–26 years	16	20.5
	27–29 years	4	5.1
	30 or more years	5	6.4
Female		93	54.4
	18–20 years	31	33.3
	21–23 years	41	44.1
	24–26 years	13	14.0
	27–29 years	6	6.5
	30 or more years	2	2.2

The bold values indicate statistically significant differences (p<0.05).

The characterization of electronic cigarette use is shown in Table 2. It was observed that the age of initiation of electronic cigarette use was related to usage settings, predominantly at parties and social gatherings. Curiosity was identified as the main motivation for electronic cigarette use, leading to personal experiences.

**Table 2:** Characterization of electronic cigarette use. Guanambi/BA, 2024 (n=171).

Variables		n	%
Age of initiation of use			
	13–15 years	3	1.7
	16–18 years	40	23.4
	19–25 years	103	60.3
	26 or more years	12	7.0
	Does not remember	5	2.9
	Did not respond	8	4.7
Frequency of use			
	Has used, but no longer uses	14	8.2
	Only tried, but did not use afterward	68	39.8
	Uses several times a day	34	19.9
	Uses several times a week	14	8.2
	Uses rarely	38	22.2
	Did not respond	3	1.7
Place of use			
	At home	4	2.4
	At parties/socializing environments	69	40.3
	No longer uses	93	54.4
	Did not respond	5	2.9
Motivations for use*			
	Curiosity	91	53.2
	It is trendy	10	5.8
	Enjoy the sensation	46	26.9
	Enjoy the flavor	47	27.5
	Influence of friends	39	22.8
	Influence of social media	7	4.1
	Socializing in certain environments	31	18.1
	Calms/relieves stress	31	18.1
	To quit smoking traditional cigarettes	7	4.1
	Perception that using it is attractive	4	2.3
	Convenience of use compared to other addictions	9	5.3
	Did not respond	5	2.9
Source of information			
	Scientific literature	18	10.5
	Social media	35	20.5
	Opinions of healthcare professionals	17	9.9
	Others	8	4.7
	Personal experiences	91	53.2
	Did not respond	2	1.2

* Multiple answers possible.

The association studies between electronic cigarette use and perceptions and beliefs are shown in Table 3. Individuals in the sample who used electronic cigarettes believed that they were more socially acceptable than conventional cigarettes (p=0.003) and that they could be used as a tool for smoking cessation (p=0.002).

**Table 3:** Perceptions, beliefs, and electronic cigarette use. Guanambi/BA, 2024.

Variables		Electronic cigarette use			
		Yes	No	Total	p-value
		n (%)	n (%)	n (%)	
Compared to conventional cigarettes, electronic cigarettes are more socially accepted (n=410)					
	I agree	161 (41.4)	228 (58.6)	389 (94.9)	**0.003**
	I disagree	2 (9.5)	19 (90.5)	21 (5.1)	
The flavors used in electronic cigarettes cause harm to health (n=308)					
	I agree	101 (37.0)	172 (63.0)	273 (88.6)	0.500
	I disagree	15 (42.9)	20 (57.1)	35 (11.4)	
Electronic cigarettes can be used as a tool for smoking cessation (n=371)					
	I agree	51 (50.5)	50 (49.4)	101 (27.2)	**0.002**
	I disagree	89 (33.0)	181 (67.0)	270 (72.8)	
Electronic cigarettes can act as a gateway to the use of conventional cigarettes (n=399)					
	I agree	117 (34.1)	226 (65.9)	343 (86.0)	**<0.001**
	I disagree	34 (60.7)	22 (39.3)	56 (14.0)	
The use of electronic cigarettes increases the risk of cancer (n=392)					
	I agree	151 (85.0)	239 (15.0)	390 (99.5)	0.080
	I disagree	2 (100.0)	–	2 (0.5)	
The use of electronic cigarettes increases the risk of neurological disease (n=315)					
	I agree	103 (33.8)	202 (66.2)	305 (96.8)	**0.003**
	I disagree	8 (80.0)	2 (20.0)	10 (3.2)	
The use of electronic cigarettes increases the risk of cardiovascular disease (n=375)					
	I agree	144 (38.7)	228 (61.3)	372 (99.2)	0.850
	I disagree	1 (33.3)	2 (66.7)	3 (0.8)	
The use of electronic cigarettes increases the risk of lung disease (n=414)					
	I agree	159 (38.5)	254 (61.5)	413 (99.7)	0.430
	I disagree	–	1 (100.0)	1 (0.3)	

Statistically significant values are denoted in bold.

On the other hand, individuals in the sample who used electronic cigarettes disagreed that these devices could act as a gateway to conventional cigarette use (p<0.001) and that they increased the risk of neurological diseases (p=0.003) (Table 3).

## DISCUSSION

In the present study, approximately 39% of university students reported using electronic cigarettes. This finding is relevant when compared to international studies, which show significant variation in the prevalence of use among young people. In several countries, a higher tendency of use is observed among boys aged 11–17 years, with no statistically significant differences regarding age, school grade, or financial resources^12^. Another study involving young people aged 18–30 years found no association between gender and the degree of electronic cigarette dependence, indicating a homogeneous distribution between men and women across all levels of dependence^13^.

The initiation of electronic cigarette use was associated with socialization settings, such as parties, where peer influence plays a crucial role. According to the study by Zhao et al.^14^, the initiation of electronic cigarette use generally occurs during adolescence, motivated by curiosity and the desire to replace traditional cigarettes. Additionally, factors such as peer pressure, lack of knowledge about risks, and exposure to advertising in stores and on social media encourage use among young people.

The high prevalence of electronic cigarette use among young adults aged 18–24 years, including current smokers, reflects a complex interplay of age, motivations, and social influences^15^. These influences include the perception that electronic cigarettes are less harmful than conventional cigarettes, a belief supported by the idea that they contain fewer harmful chemicals^16,17^. Exposure to electronic cigarette advertising, whether online or through traditional channels, is strongly associated with a higher perceived social acceptability of their use^18^.

A crucial factor contributing to the initiation and continuation of electronic cigarette use is the wide variety of available flavors. These flavors make vaping more appealing to adolescents and young adults, despite evidence linking flavored aerosols to impaired lung function, leading to respiratory problems^19,20^. The combination of these misconceptions and risk factors underscores the need for greater public awareness of the risks associated with vaping.

The results of this study corroborate the growing consensus that electronic cigarette use is associated with an elevated risk of various health problems, including cancer, as well as cardiovascular, neurological, and pulmonary diseases. Harmful substances present in electronic cigarettes can irritate the respiratory tract, cause pulmonary diseases, and contribute to neurological disorders. Furthermore, the use of these devices may increase the risk of cardiovascular diseases, such as myocardial infarction and stroke, especially in individuals who have never smoked traditional cigarettes^21,22^.

Evidence also indicates that electronic cigarettes contribute to endothelial dysfunction and harmful changes in blood lipid profiles, which are crucial factors for cardiovascular health^23^. This growing body of evidence highlights the need for more intense public awareness and strict regulatory measures to mitigate the dangers associated with electronic cigarette use^24^.

In the USA, studies indicate that sexual minority youth, particularly Black gay, lesbian, and bisexual individuals, are more likely to use electronic cigarettes compared to their heterosexual peers^25^. This points to an additional vulnerability that needs to be considered in prevention strategies.

This study was conducted in a single municipality in the interior of Bahia, which may limit the generalizability of the results, as factors such as access to devices, regulatory enforcement, and sociocultural contexts vary across regions. This may have influenced students’ perceptions and behaviors, reflecting a specific local scenario. Additionally, the face-to-face application of the questionnaire may have introduced social desirability bias in the responses.

Despite these limitations, the findings have important practical implications. The high prevalence of use and misconceptions about the risks of electronic cigarettes highlight the need for more effective educational campaigns, stricter regulations on advertising and sales, and intersectoral actions aimed at prevention and awareness among university students. Future studies with national coverage and qualitative methods are recommended to deepen these findings.

These findings underscore the importance of stricter regulations, targeted awareness campaigns, and additional research to correct common misconceptions. Many young people mistakenly believe that electronic cigarettes are less harmful than conventional cigarettes and do not contain harmful substances. However, these devices release dangerous chemical compounds. Additionally, the belief that electronic cigarette use is a safe alternative to traditional smoking, without understanding the risks associated with prolonged use, represents a significant public health challenge. Therefore, promoting regulatory and educational measures is essential to protect young people's health and mitigate the risks associated with electronic cigarette use.

## CONCLUSION

The prevalence of electronic cigarette use among university students was found to be 39.2%, with no statistically significant differences between genders. Curiosity was identified as the main motivation for electronic cigarette use, leading to various personal experiences. Individuals who use electronic cigarettes believe that they are more socially acceptable than conventional cigarettes and can be used as a tool for smoking cessation. On the other hand, these individuals disagree that electronic cigarettes could serve as a gateway to conventional cigarette use or increase the risk of neurological diseases.

These findings indicate a significant prevalence of electronic cigarette use among university students, with varied perceptions regarding its risks and benefits. It is crucial to continue monitoring these trends and to develop health education strategies to address misconceptions and promote the well-being of young people.

## Data Availability

The datasets generated and/or analyzed during the current study are available from the corresponding author upon reasonable request.
